# The Effectiveness of Exercise Therapy on Scapular Position and Motion in Individuals With Scapular Dyskinesis: Systematic Review Protocol

**DOI:** 10.2196/resprot.8011

**Published:** 2017-12-13

**Authors:** Afsun Nodehi Moghadam, Kianoush Abdi, Mohsen Shati, Shohreh Noorizadeh Dehkordi, Abbas Ali Keshtkar, Zahra Mosallanezhad

**Affiliations:** ^1^ Department of Physiotherapy University of Social Welfare and Rehabilitation Sciences Tehran Islamic Republic Of Iran; ^2^ Department of Rehabilitation Management University of Social Welfare and Rehabilitation Sciences Tehran Islamic Republic Of Iran; ^3^ Department of Aging University of Social Welfare and Rehabilitation Sciences Tehran Islamic Republic Of Iran; ^4^ Department of Physiotherapy School of Rehabilitation Sciences Iran University of Medical Sciences Tehran Islamic Republic Of Iran; ^5^ Department of Health Sciences Education Development School of Public Health Tehran University of Medical Sciences Tehran Islamic Republic Of Iran

**Keywords:** scapula, exercise therapy, rehabilitation, systematic review, protocol

## Abstract

**Background:**

Scapular dyskinesis is an alteration in normal scapular position and motion. Some researchers believe that altered kinematics of the scapula subsequent to dysfunction or weakness of scapular stabilizing muscles contributes to impingement syndrome. Scapular muscle exercises are included in the rehabilitation of patients with subacromial impingement syndrome and scapular dyskinesis because the muscular system is one of the major contributors of scapular positioning both at rest and during shoulder movement, but there is considerable uncertainty relating to the relative effectiveness of such approaches on changing scapular position and motion.

**Objective:**

The aim of this systematic review protocol is to evaluate the effectiveness of exercise therapy on scapular position and motion in individuals with scapular dyskinesis.

**Methods:**

A systematic review will be conducted using PubMed, Scopus, Web of Science, Elsevier, Ovid, ProQuest, Physiotherapy Evidence Database, and Cochrane Library. The reference lists of articles, other reviews, gray literature, and key journals will be searched for relevant articles. Clinical trials reporting the effect of therapeutic exercises (scapular strengthening exercise, scapular stabilization exercise, scapular muscle stretching) with the aims of changing scapular position and motion in individuals with scapular dyskinesis will be included. Two independent reviewers will select studies, extract data, and assess the quality of primary studies. Any disagreement during the selection of studies will be discussed and decided by the whole team.

**Results:**

This systematic review began in December 2016 and is currently in progress. The findings will be synthesized to determine the effectiveness of recommended therapeutic exercise on scapular position and motion in individuals with scapular dyskinesis.

**Conclusions:**

This is the first systematic review protocol aiming to assess the effectiveness of exercise therapy in individuals with scapular dyskinesis. The systematic review doesn’t require ethics approval because all data used will be provided from published documents. The results of this study will be published in a peer-reviewed journal.

**Trial Registration:**

PROSPERO CRD42017053923; https://www.crd.york.ac.uk/prospero/display_record.php?RecordID=53923 (Archived by WebCite at http://www.webcitation.org/6uzq32T02)

## Introduction

### Background

Elevation of the arm requires normal function of the rotator cuff to stabilize the humeral head in the glenoid fossa and coordinated motion of the scapula [1]. During arm elevation, the scapula upwardly rotates, posteriorly tilts, and externally rotates [2,3]. These scapular motions depend on normal function of the scapular stabilizers including trapezius, rhomboid, and serratus anterior muscles [1].

Scapular dyskinesis is an alteration in normal scapular position and motion. It is characterized by prominence of the scapular medial border and/or inferior angle relative to the thoracic cage in the static position or in dynamic motion; early scapula elevation or shrugging during arm elevation; or inadequate upward and downward rotation of the scapula during arm elevation or lowering [4,5]. Scapular dyskinesis was found to be present in 61% of overhead athletes and 33% of nonoverhead athletes [6]. It has also been revealed in 67% to 100% of athletes with shoulder injuries and also in many asymptomatic athletes [6,7]. Scapular dyskinesia or altered kinematics of the scapula (downward rotation, anterior tilt, and internal rotation) subsequent to dysfunction or weakness of scapular stabilizing muscles may contribute to impingement syndrome through decreasing the subacromial space [8-10].

Subacromial impingement syndrome and rotator cuff tendinopathy account for 44% to 65% of all complaints of musculoskeletal shoulder pain, and they have been linked to scapular dyskinesis [11,12]. The researchers have suggested that scapular position and movements are altered in patients with subacromial impingement syndrome [8-10], rotator cuff tendinopathy [13], shoulder instability [14,15], and even neck pain [16]. It is not clear that the association between scapular dyskinesis and shoulder pathology represents a cause or effect of the pathology [6,17,18].

The clinical evaluation of scapular motion is challenging because of the 3-dimensional scapular motion. Previous studies have described different methods of identifying scapular dyskinesis including an electromagnetic kinematic motion analysis system, 3-dimensional digitizer, visual observation, linear measurement, and manual correction maneuvers [19-22].

Exercise is a key component of shoulder rehabilitation. A systematic review by Hanratty et al [23] showed that physiotherapy exercises are effective in decreasing pain and improving function in patients with subacromial impingement syndrome at short-term follow-up. Scapular muscle exercises are included in the rehabilitation of patients with subacromial impingement syndrome and scapular dyskinesis because the muscular system is one of the major contributors of scapular positioning both at rest and during shoulder movements [24], but there is considerable uncertainty relating to the relative effectiveness of such approaches on changing scapular position and motion.

A recent systematic review and meta-analysis of 4 randomized clinical trials (RCTs) in patients with rotator cuff disorders concluded that a scapula-focused approach is more effective than generalized approaches in reducing shoulder pain up to 6 weeks, but this benefit is not apparent by 3 months. This systematic review also showed a conflicting result regarding the effect of a scapula-focused approach in comparison to generalized approaches on scapula position and movement [25]. In the aforementioned systematic review, the primary objective was to synthesize the impact of scapular intervention on rotator cuff–related shoulder pain, with the secondary objective being evaluating the effect of scapular exercises on scapula position and movement; however, it did not include gray literature, probably missing some related studies. There are some studies in which changing scapula kinematics in asymptomatic individuals has been studied.

Previous studies have shown scapular dyskinesis to be detrimental to shoulder function, and they recommend improvement or correction of abnormal scapular mechanics [6,22,26]. The correction of scapular dyskinesis has been recommended for decreasing the symptoms associated with shoulder pathology [6,22,26], but the effects of scapular dyskinesis interventions on correcting scapular position and motion is not clear yet. Therefore, the main aim of this systematic review will be the evaluation of the effectiveness of exercise therapy on scapular position and motion in individuals with scapular dyskinesis.

### Objectives

#### Primary Objective

- Identify the effectiveness of exercise therapy (scapular strengthening exercise, scapular stabilization exercise, scapular muscle stretching) on scapular position and motion of individuals with scapular dyskinesis

#### Secondary Objectives

- Identify the effectiveness of scapular exercise on shoulder pain in rotator cuff disorder patients with scapular dyskinesis
- Identify the effectiveness of scapular exercise on changing scapular kinematics in asymptomatic individuals with scapular dyskinesis
- Identify the effectiveness of scapular exercise on scapular position and motion of individuals with different types of scapular dyskinesis
- Find potential sources of heterogeneity in primary studies

## Methods

### Study Characteristics

This systematic review will include any types of clinical trials (RCTs with or without concurrent control; double blind, single blind, and open-label RCTs; and before-after clinical trials) in which scapular position or motion is considered one of the main independent variables. Case studies and simulation studies will not be included.

### Types of Participants

This systematic review will include studies with adult participants (aged 16 years and older, athletes and nonathletes) in which a clear diagnosis of scapular dyskinesis is defined according to any of following criteria:

#### Abnormalities in Scapular Rest Position

- Scapular winging: prominence of scapular medial border and/or inferior angle relative to the thoracic cage
- Scapular tilt or protraction
- SICK scapula syndrome: scapular malposition, inferior medial border prominence, coracoid pain, malposition and dyskinesis of scapular movement

#### Abnormalities in Scapular Motion

- Scapular dysrhythmia: early scapula elevation or shrugging during arm elevation and inadequate upward and downward rotation of the scapula during arm elevation and lowering (scapular downward rotation syndrome) [22]

The studies that focused on the effects of changing scapular kinematics in asymptomatic individuals or individuals with rotator cuff tendinopathy and subacromial impingement syndrome will be included. The studies without a clear diagnostic criterion and clinical measures for scapular dyskinesis will be excluded.

### Types of Intervention

The intervention in the treatment group should be scapular focused exercises with or without general shoulder exercise. The control group will include other forms of interventions, such as manual therapy and taping, or no treatment.

Studies reporting any type of therapeutic intervention (scapular strengthening exercise, scapular stabilization exercise, scapular muscle stretching) with the aims of changing scapular biomechanics, including position and movement, and addressing the pain and disability found with scapular dyskinesis will be included. Also, scapular exercise combined with patient education and instruction on exercises will be included. Clinical trials that compare scapular kinematics after using other techniques such as manual therapy and taping and studies in which exercise has been a minor component of a multimodal approach will be excluded.

### Outcome Assessment

The primary outcome will be measurements reported on scapular kinematics outcomes such as scapular rest position, static scapular positioning, scapulohumeral rhythm, and scapular dynamic control (eg, lateral scapular slide test, measurement of scapular upward and downward rotation, measurement of scapular anterior and posterior tilt, and measurement of scapular medial and lateral tilt). Our secondary outcome will include shoulder pain intensity.

### Information Sources

Our electronic search database includes PubMed, Scopus, Web of Science, Elsevier, Ovid, ProQuest, Physiotherapy Evidence Database, and the Cochrane Library. The reference lists of articles, other reviews, gray literature, and key journals will be also searched for relevant articles. All studies will enter the initial screening stage without any time limit or restrictions of language and publication type.

### Search Strategy

The strategy for searching will be developed and completed in the PubMed database, and then the same strategy will be applied to the other electronic databases. Textbox 1 shows the suggested PubMed search strategy.

### Study Records

Two authors will search information sources independently and perform the primary article screening. At first, they will screen the titles and abstracts of all the articles independently, and then their selected articles will be categorized as eligible, not eligible, or may be eligible. When a study cannot be clearly excluded according to its title and abstract alone, its full text will be reviewed before the final decision. Articles categorized as not eligible by both reviewers will be eliminated. Each reviewer will then review the full text of the remaining articles, and a study will be included when both reviewers independently assess it as satisfying the eligibility criteria. Any disagreement during the selection of studies will be discussed and decided by the whole team.

### Data Extraction and Management

Data will be extracted from papers by 2 reviewers and entered into a data extraction form. Any disagreement will be discussed and decided on by the whole team. If there are incomplete or unclear data in articles, inquiries will be sent to the authors. All searched studies will be managed through EndNote (Clarivate Analytics) software.

### Data Items

From each article, the following information will be extracted: general information (author name, year of publication, journal title, date of extraction), study characteristics (title of study, study design, study setting, sample size), participant characteristics (demographics data, main inclusion criteria, sports history, healthy condition, and type of scapular dyskinesis), methods and tools of scapular dyskinesis diagnosis (observational, 2- and 3-dimensional assessment methods), intervention characteristics (exercise type, frequency, and duration), scapular outcome measurements and results (eg, lateral scapular slide test, measurement of scapular upward and downward rotation, measurement of scapular anterior and posterior tilt, and measurement of scapular medial and lateral tilt), and pain outcome measure.

**Suggested PubMed search strategy.**

1. Scapular dyskinesis
2. Scapular orientation
3. Scapular position
4. Scapular winging
5. Scapular protraction
6. Scapular malposition
7. SICK scapula (SICK: scapular malposition, inferior medial border prominence, coracoid pain, and abnormal movement of the scapula)
8. SICK scapular syndrome
9. Scapula* dyskines*
10. Scapular posterior tilt
11. Scapular posterior tipping
12. Scapular anterior tilt
13. Scapular anterior tipping
14. Scapular upward rotation
15. Scapular downward rotation syndrome
16. Scapulothoracic*
17. Scapulohumeral rhythm
18. Scapular kinematics
19. Scapular dysrhythmia
20. Rotator cuff tendinopathy
21. Shoulder impingement syndrome
22. Subacromial impingement syndrome
23. Scapula* exercise*
24. Scapular stabilization exercises
25. Scapula* stabili*
26. Shoulder rehab*
27. Shoulder stretch*
28. Shoulders strengthen*
29. Shoulder exercise*
30. Scapular approaches
31. 1 or 2 or 3 or 4 or 5 or 6 or 7 or 8 or 9 or 10 or 11 or 12 or 13 or 14 or 15 or 16 or 17 or 18 or 19 or 20 or 21 or 22
32. 23 or 24 or 25 or 26 or 27 or 28 or 29 or 30
33. 31 and 32

### Assessment of Risk of Bias in Included Studies

The risk of bias assessment will be based on the Cochrane Collaboration Risk of Bias [27]. Risk of bias in each study will be independently assessed by 2 reviewers. Each reviewer will evaluate methodological quality using the following items: random sequence generation, allocation concealment, blinding of participants and personnel, blinding of outcome assessment, incomplete outcome data, selective reporting, and other bias. After critical appraisal of studies, each trial will be categorized into low risk, high risk, and unclear risk. In case of disagreement, discussion will take place to achieve consensus.

### Data Synthesis

If enough studies are included, a meta-analysis will be conducted using the scapula and pain outcome data. For scapular outcome data, mean differences and standardized mean differences will be used for meta-analysis. In cases where data are missing, they (eg, standard deviation) will be calculated from available data (eg, standard error will be calculated from *P* values or 95% confidence intervals) [28] or we will contact the authors.

If sufficient comparable studies are included, a subgroup analysis will be carrying out. The subgroups will be formed by quality of primary studies, type of study participants (athlete and nonathlete studies and asymptomatic individuals or individuals with rotator cuff–related shoulder pain), and types of scapular dyskinesis (scapular outcomes measurements and results).

## Results

This systematic review began in December 2016 and is currently in progress. This is the first systematic review protocol aiming to assess the effectiveness of exercise therapy in individuals with scapular dyskinesis. The findings of this systematic review will be synthesized to determine the effectiveness of recommended therapeutic exercise on scapular position and motion in individuals with scapular dyskinesis. On completion of this project, the results of this study will be published in a peer-reviewed journal.

## Discussion

Scapular dyskinesis is a condition that is commonly associated with shoulder pathology but is also present in asymptomatic individuals, and it is believed to be a risk factor for further injury [6]. Evidence suggests that patients with rotator cuff–related shoulder pain present scapular kinematic abnormalities such as decreased scapular upward rotation, decreased scapular posterior tipping, and external rotation [8,29,30]. It has been proposed that abnormal scapular kinematics may be linked to weakness of scapular muscles [31-33]. Specifically, increased activation of the upper trapezius with decreased activation of the lower trapezius and serratus anterior has been proposed to be related to altered scapular position and motion [34]. However, it is not clear if these differences are compensatory strategies or causative factors [6,17,18].

Conservative treatment, including exercise therapy, is thought to influence various shoulder conditions and outcomes such as pain, restricted range of motion, and functional disability [23,24], but there is considerable uncertainty relating to the relative effectiveness of such approaches on changing scapular position and motion. The results of this systematic review can help clinicians relating to effectiveness of therapeutic exercise on scapular dyskinesis and associated shoulder pain.

## Abbreviations

RCT: randomized clinical trial
SICK: scapular malposition, inferior medial border prominence, coracoid pain, and abnormal movement of the scapula

## Appendix

Multimedia Appendix 1 Peer-review report.
