# Supplementary material for: The Effectiveness of Exercise Therapy on Scapular Position and Motion in Individuals With Scapular Dyskinesis: Systematic Review Protocol
Source: JMIR Res Protoc. 2017 Dec 13;6(12):e240. doi: 10.2196/resprot.8011 (PMC5745349; doi:10.2196/resprot.8011)
Supplement: Multimedia Appendix 1 [file resprot_v6i12e240_app1.pdf]

Reviewers' comments and authors response

Proposal number: 1380

Proposal title: The effectiveness of exercise therapy on scapular position and motion in individuals with scapular dyskinesis: a systematic review protocol

Dear reviewers

We have carefully reviewed the comments and have revised the manuscript accordingly. Our responses are given in a point-by point manner below. Changes to the manuscript are shown in red

Review ID: A-1380

Reviewer comments # 1) you should define primary and secondary aims separately

Our systematic review has primary and secondary aims. As you suggested the primary and secondary aims defined separately.

Reviewer comments # 2) Because of high prevalence of scapular dyskinesis in athletes; please exactly determine which subjects will be included in your study?

According to our inclusion criteria we are going to include athletes and non-athletes studies. If sufficient comparable studies are included, the subgroup analysis will be carrying out.

Reviewer comments # 3) In the outcome assessment section you just point to scapular kinematics as your study outcome, it is better if you have more outcomes assessment , you describe and divide them as primary and secondary outcomes

It was added

Review ID: B-1380

Reviewer comments # 1) At the end of introduction part you have cited to a similar systematic review, please explain exactly what is the gap of this study and what are you going to add to their study?

We explain it with more detail. These sentences were added to introduction section.
In mentioned systematic review the primary objective was to synthesize the impact of scapular intervention on rotator cuff related shoulder pain and gray literature wasn't included in their study.

Reviewer comments # 2) the most of recent systematic reviews have primary and secondary objectives and outcome measures, if you have, please explain them in details.

Our systematic review has primary and secondary aims. The primary and secondary aims defined separately.

Reviewer comments # 3) At least one example of your search strategy should be added in detail

The strategy for searching the PUBMED database was added as you suggested

Reviewer comments # 4) in data extraction section; please mention what data will be extracted

It was added. It was written in manuscript as Data items title
